# “Maze Out”: a study protocol for a randomised controlled trial using a mix methods approach exploring the potential and examining the effectiveness of a serious game in the treatment of eating disorders

**DOI:** 10.1186/s40337-024-00985-2

**Published:** 2024-03-01

**Authors:** Maria Mercedes Guala, Aida Bikic, Kim Bul, David Clinton, Anna Mejldal, Helene Nygaard Nielsen, Elsebeth Stenager, Anette Søgaard Nielsen

**Affiliations:** 1 Psychiatric Research Unit, Institute of Clinical Research, University of Southern Denmark, J.B. Winsløws Vej 18, 5000 Odense, Denmark; 2 Department of Regional Health Research, Faculty of Health, University of Southern Denmark, Odense, Denmark; 3 Centre for Intelligent Healthcare, Coventry University, Coventry, UK; 4 Department of Medical Epidemiology and Biostatistics (MEB), Centre for Eating Disorders Innovation (CEDI), Karolinska Institute, Stockholm, Sweden; 5 Open Patient Data Explorative Network, Odense University Hospital, Odense, Denmark; 6 Child and Adolescent Psychiatric Services Southern Jutland, Region of Southern Denmark, Aabenraa, Denmark

**Keywords:** Eating disorders, Serious games, Self-efficacy, Co-production, Randomised controlled trial

## Abstract

**Background:**

Eating Disorders (ED) are severe and costly mental health disorders. The effects of existing treatment approaches are limited and there is a need to develop novel interventions, including digital strategies that can increase engagement and effectiveness. Maze Out is a new serious game coproduced by patients and ED therapists, which allows patients to “play” with the reality of an ED and reflect on associated challenges.

**Objectives:**

The present study has two main objectives: (1) to evaluate the effectiveness of adding Maze Out to treatment as usual (TAU) in a randomised controlled trial (RCT); and (2) to examine in depth the potential of Maze Out by examining how it is perceived and used in the context of an RCT.

**Methods:**

Participants will be recruited from mental health care services, endocrinology departments or Community Centres offering treatment for ED. Patients suffering from ED (N = 94) will be randomised to either TAU or TAU plus Maze Out. Primary outcome will be measured in terms of changes in self-efficacy, measured by a 5-item self-efficacy questionnaire (5-item SE_ED). Secondary outcome measures will include feelings of ineffectiveness and self-image, as measured by Eating Disorder Inventory, version 3 (EDI-3), Brief INSPIRE-O and Structural Analysis of Social Behaviour Intrex Questionnaire (SAS-B). Data will be collected at baseline (enrolment in the study), and subsequently 8 and 15 weeks after inclusion. Experiences of playing Maze Out will be examined in a sub-sample of participants, utilising both quantitative user analytics and qualitative interview data of patients, interview data of significant others, and healthcare professionals to explore the possible impact of Maze Out on disorder insight, communication patterns between patients and therapists and understanding of their disorder.

**Discussion:**

To our knowledge Maze Out is the first serious game coproduced by patients and therapists. It is a novel and theoretically grounded intervention that may significantly contribute to the healing process of ED. If found effective, the potential for wide-spread impact and scalability is considerable.

*Trial registration* ClinicalTrials.gov NCT05621018.

**Supplementary Information:**

The online version contains supplementary material available at https://doi.org/10.1186/s40337-024-00985-2.

## Background

Eating disorders (EDs) are severe mental health disorders with high mortality, in addition to being costly and associated with serious impairments in quality of life [1–4]. The lifetime prevalence of EDs in Western countries is high with rates of 1.89% across the general population and 2.58% among females [5]. Currently, the recommended treatment for ED are different forms for evidence-based treatments such as enhanced cognitive behavioral therapy (CBT-E) for eating disorders [6, 7], The Maudsley Model of Anorexia Nervosa Treatment for Adults (MANTRA) [8], focal psychodynamic therapy (FPT) [9] mentalisation-based treatment for eating disorders (MBT-ED) [10], and for binge eating disorder (BED) also psychopharmacological treatment [11–16]. The clinical presentation of EDs is complex and depends on both the duration and severity of the disorder, as well as the life circumstances of the patient. These are probably some of the reasons why effective evidence-based treatment of ED is limited [6–9]. Moreover, challenges for healthcare staff include the patients’ lack of insight into their own disorder, high dropout rates, ambivalence attitude towards recovery, and poor treatment alliance [10, 17–19]. Individuals with an ED often do not identify themselves with a diagnostic label or as having a mental disorder [20]. Ambivalence to recovery and lack of insight into their disorder can partly be explained by the ego-syntonic aspects of EDs. Simultaneously, many patients with EDs develop a sense of helplessness and hopelessness. In such cases, the ambivalence towards treatment may centre around having a need to recover from the ED, but at the same time not having a sense of agency, or feeling unable to do anything to improve [21].

Self-efficacy is an individual’s belief in his or her capacity to execute behaviours necessary to produce specific performance attainments [22]. EDs are associated with low self-efficacy and high perfectionism [23, 24]. Furthermore, self-efficacy has shown a negative significant relationship with perfectionism in patients with ED, often increasing its symptoms [25]. Self-efficacy has also been shown to be a robust predictor of outcomes in several other psychiatric disorders, is an important predictor of outcomes in BED [26] as well as outcomes in underweight ED patients such as short-term hospital treatment [23]. There is thus a need to develop effective and engaging interventions that can enhance patients’ self-efficacy, stimulate insight into their disorder, and strengthen collaboration with clinicians and significant others such as family members, partners and friends [16, 27–29].

A pilot study of user experience and acceptance of the serious game Maze Out suggests that an engaging serious digital game that can augment treatment may improve patients’ self-perceived ability to create change and insight [29]. Serious Games (SGs) are defined as “digital games created not with the primary purpose of pure entertainment, but with the intention of serious use as in training, education and health care” [30]. SGs are gaining a greater evidence-base in both somatic and mental health care contexts [29, 31–34], probably due to their around-the-clock availability, their potential to motivate, and their ability to engage users in a challenging problem while exploring new solutions without experiencing real-life risks. Developing such games for EDs may help patients meet and cope with the challenges of their problems, specifically through their potential for changing perceptions and stimulating insight. However, so far, SGs have only been implemented to a very limited extend in the treatment of EDs. A few studies have focused on serious video games and Virtual Reality (VR) as potential digital interventions; these have been used as therapeutic adjuncts and show promise in terms of improving outcomes such as emotional regulation, body dissatisfaction, and eating disorders symptoms [35–38]. To our knowledge, Maze Out is the first SG developed and piloted to support people with EDs and can be played on a tablet or smartphone, making them easily available. Maze Out was co-produced by patients with EDs themselves which also makes it unique. Until now, only one other study has used experience-based co-design to develop an intervention for patients with EDs; a cognitive behavioural therapy-based intervention for people with type 1 diabetes and disordered eating [39].

### Maze Out

Maze Out is an SG that can be played on a tablet or smartphone [29]. The game is accessible via the domain name and the username has to be activated by the researchers before the participant can use it. It is free of charge for the participants. The costs incurred for operating the game (about US $800 per 1.000 patients per annum) are covered by the health service. The game is intended to provide an additional therapeutic component to treatment as usual (TAU) [29]. Maze Out was coproduced at the Psychiatric Hospital in the Region of Southern Denmark from January to December 2020 in close collaboration with four patients with different ED diagnoses (anorexia nervosa, atypical anorexia nervosa, bulimia nervosa and eating disorder not otherwise specified) (Additional file 1: Appendix 1), three therapists, and a commercial game company experienced in developing educational games. To our knowledge, Maze Out is the first SG produced jointly by patients with EDs and therapists. The development of such collaborative interventions is rare in the field of EDs [40], but essential when developing need-drive, feasible and relevant content, as well as technology that can be successfully integrated into the existing treatment of patients [16].

Maze Out is built around a narrative, which presents the player with a scenario of being caught in a dream. In this dream the player finds him or herself in the middle of a maze from which the player can only escape by making decisions. On the journey out of the maze the player is faced with fifteen missions that need to be resolved. These missions relate to challenges in everyday life for someone with an ED and focus on relationships, feelings, and bodily experiences. There are no right or wrong decisions, but the decisions have congruent consequences. During the game the player is regularly invited to pause and tune in with his/her feelings and bodily sensations. At the end of every mission a therapist character within the game invites the player to reflect on his/her feelings and reactions that may have arisen while playing and register their answer in the game. An example of a mission (“Say what you think”) can be found in the Additional file 2: Appendix 2. Here the player is invited to her/his mother’s birthday but does not feel that she/he has the mental energy to go. This mission challenges the player’s ability to rely on feelings and bodily sensation to make a decision. The player is invited to experience stress or “chaos” about having opposite feelings in a “safe environment” and inspired to talk about it with others.

The current protocol describes the strategy for evaluating the effectiveness of Maze Out and exploring how it is perceived by patients, clinicians, and significant others.

### Theoretical underpinnings of Maze Out

One of the fundamental theoretical foundations of Maze Out stems from Hilde Bruch (1904–1984), a German-born American psychiatrist and psychoanalyst who is probably the most influential figure in the field of EDs [41]. She describes the main psychopathological phenomena of EDs as the lack of awareness of inner experiences and failure to rely on feelings, thoughts, and bodily sensations to guide behaviour. This may contribute to the experience of not living one’s own life [42] and help to explain why many individuals with EDs describe an overwhelming experience of stress and inner restlessness or “chaos” [43]. Accordingly, ED symptoms can be understood as depositaries of symbolic meaning, encompassing mental representations and processes that regulate affect and alleviate painful inner states [44, 45]. This theory has had considerable influence in understanding the function of EDs [10, 44, 46, 47].

Another important theoretical foundation for the coproduction of Maze Out is to be found in contemporary cognitive models of EDs, which propose that rigidity, focus on detail, and social-emotional difficulties play a role in the development and maintenance of the disorder [48–50]. From this perspective, exclusive focus on the body and food is associated with an emotionally numbing experience and increased avoidance of social interactions that are seen as increasingly threatening and intolerable due to their potential for conflict, criticism, and the activation of negative emotions. According to such models, working on decreasing emotional avoidance and reducing cognitive rigidity can be a salient way of reducing ED symptoms [51].

From a theoretical perspective, the notion of play is of importance in SGs in general and Maze Out in particular. Playing can be seen as more than an activity for enjoyment and recreation since it also fulfils important psychological and social functions. From a developmental perspective, playing allows children to experiment with their behavioural and social repertoire, as well as practice their physical and communication skills [52]. The same could be said of adults when they engage in play. In both children and adults, playing can be considered as a means of exploring things that are both wished for and feared. Playing confronts the challenges of the living by inscribing and sustaining an imaginative dimension [53].

These theoretical foundations provide the basis for the approach of Maze Out as a SG and the EDs symptoms that this SG aims to address. To ensure that Maze Out was designed in a way that is meaningful and attractive to patients, it was decided that the development of Maze Out would be with the participation of patients and clinicians in a coproduction framework. This is also to ensure that the exercises and tasks within the task are understandable and invite reflection. The Maze Out pilot study indicates that the SG successfully accomplishes the latter [29].

### Aims

The aims of the present study are twofold: (1) to evaluate the effectiveness of Maze Out when added to ED treatment as usual (TAU) in a randomised controlled trial (RCT); and (2) to explore the experience and use of Maze Out within context of the RCT.

At the end of this study, we expect to be able to know whether Maze Out can be recommended as a supplementary tool for treatment and if so, describe which type of patients would be able to benefit from this.

### Hypotheses

We hypothesize that that the addition of Maze Out to TAU for EDs will enhance the self-efficacy of participants as well as reduce feelings of ineffectiveness and insecurity, as well as increase patients’ confidence in their ability to deal with physical and emotional limitations. We also hypothesize that Maze Out will reduce patients’ interpersonal problems, expressed in terms of general inadequacy, insecurity, worthlessness and negative self-evaluation (i.e. self-concept) [54].

On average, the minimum length of ED treatment (i.e. TAU) in Denmark is 15 weeks [55], therefore we assume that Maze Out will need to be play at least for 15 weeks for showing any impact on patients.

Therefore, the primary and secondary research questions of this study are as follows:

- *Primary* Does Maze Out improve patients’ sense of self-efficacy after playing for a 15-week period compared to TAU alone?
- *Secondary* Does Maze Out have an impact on patients’ feelings of ineffectiveness and personal recovery process compared to TAU alone?

## Methods

### Design

Intervention effectiveness will be tested with a mixed-method approach by a randomised controlled trial in which change in outcomes of patients with EDs such as self-efficacy, will be compared across “Maze Out” + TAU vs. TAU alone groups. Patients who fulfil the inclusion criteria will be randomised to the two conditions. Randomisation will be conducted using the built-in randomisation module in REDCap (Research Electronic Data Capture) [56] from the Odense Patient Data Explorative Network (OPEN). To ensure adequate allocation concealment, the random allocation sequence will be generated before patient enrolment begins, by a member in the research group (RB) who is independent and not otherwise involved in the study. The researcher in charge of obtaining written informed consent will initiate the randomisation procedure when the patient has agreed to participation and completed the baseline measures. Patients will be informed of the results of randomization immediately after the procedure has been conducted. No stratification will take place. Preliminary comparisons based on stratification will be explored in order to inform future research using Maze Out.

A subgroup of patients randomised to Maze Out + TAU will be selected for qualitative interviews, taking into consideration both patients who have played the 15 intervention weeks and patients who have played a few times or not at all.

### Participants

Participants will be recruited through their contact person or clinician from a broad set of treatment institutions in Denmark, offering treatment and care to patients diagnosed with ED. The treatment institutions include psychiatric centres within the mental health care services, psychiatric clinics, municipal ED teams, mental health care institutions, and endocrinology services specialised in EDs. All regions in Denmark will be invited to participate. Recruitment from a wide range of treatment institutions and locations is intended to reflect everyday practice and nuances in clinical approach to EDs in Denmark.

To be eligible to participate, patients must: (1) agree to participate in the study and sign written informed consent; (2) be aged 18 or above; (3) speak, read and understand Danish; (4) have a registered ED diagnosis at the treatment site, according to ICD-10 and, made by at list a clinical interview and biometrics such as BMI (i.e., not only ED symptoms) and (5) receive support or treatment for ED. Exclusion criteria: There is no exclusion criteria. The ability to speak, read and understand Danish is necessary as Maze Out is currently only available in Danish.

### Treatment interventions

#### ED treatment as usual (TAU)

TAU offered at the participating units is diverse and mainly consisting of psychological treatment combined with nutritional counselling and support for everyday functioning. Psychological treatment involves elements of Cognitive Behavioural Therapy (CBT) [57], Mentalization-based treatment (MBT) [10] and physiotherapy. These methods are utilised predominantly at mental health care services and psychiatric clinics, while the nutritional counselling is the major component in endocrinology services, and support for everyday functioning is utilised in municipal ED teams. Some patients receive treatment in a group setting, while others receive individual treatment or both. Psychological treatment is delivered by trained therapists, such as psychiatric nurses, social workers and clinical psychologists. Nutritional advice is delivered by dieticians, nurses and physicians. Patients receiving TAU only will not have access to Maze Out for 15 weeks after recruiting. After 15 weeks, and after they have completed their measures, these patients will receive a link that allows them to download and play Maze Out to make participation in the study ethically fair. An online self-rapport questionnaire will be used at baseline to gain understanding of what TAU consists of (Additional file 3: Appendix 3).

#### *Maze Out* + *ED treatment as usual (TAU)*

The group allocated to this condition will receive TAU and access to play Maze Out. Patients will be asked to play at least once a week or as long as they wish for 15 weeks in total. Maze Out will be provided through a link whereby they can download the game on their own Android or Apple devices such as smartphones and tablets.

### Procedure

Patients will be informed about the study by the healthcare staff they are usually in contact with (e.g., therapist or contact person) and asked if they are willing to meet with a trained research assistant who will provide further study information. If the patient agrees, the research assistant will provide written and oral information about the study. Patients will then have at least one week to decide if they want to participate in the study, before being asked to provide written informed consent. Participants will be able to leave the study at any time without reason and without any consequences for current or future treatment. After obtaining informed consent, baseline measurements will be obtained.

### Data management

Outcomes will be assessed using information from patient case notes and self-report questionnaire (Additional file 4: Appendix 4) provided via a secure email system (e-Boks). Data from self-report questionnaires will be returned via secure routes to a secure data base (REDCap) [56]. Data collected from patients’ health records will be entered directly to REDCap by a trained research assistant. Back-end data from Maze Out will be collected pseudonymized and transferred to RedCap by a trained research assistant.

All data collected in the study will be treated as strictly confidential. Data will be anonymized (person sensitive data) and encrypted (group allocation) before extraction by an external data manager and transmitted through secure pathways to a secure data base. Researchers responsible for reporting the results will not have access to the data and will receive the analysis in their final form for reporting. A Statistical Analysis Plan (SAP) will be written before any analysis on the data will be performed, where the blinding is lifted for the statistician.

### Measurements

Data on the use of Maze Out will be collected from the back-end of Maze Out, which consists of: (a) information on which portals the patient has been through; (b) duration and frequency the patient has played the game; and (c) answers to questions within different missions of Maze Out. These data will be collected throughout the 15-week period patients play Maze Out.

**Fig. 1:**
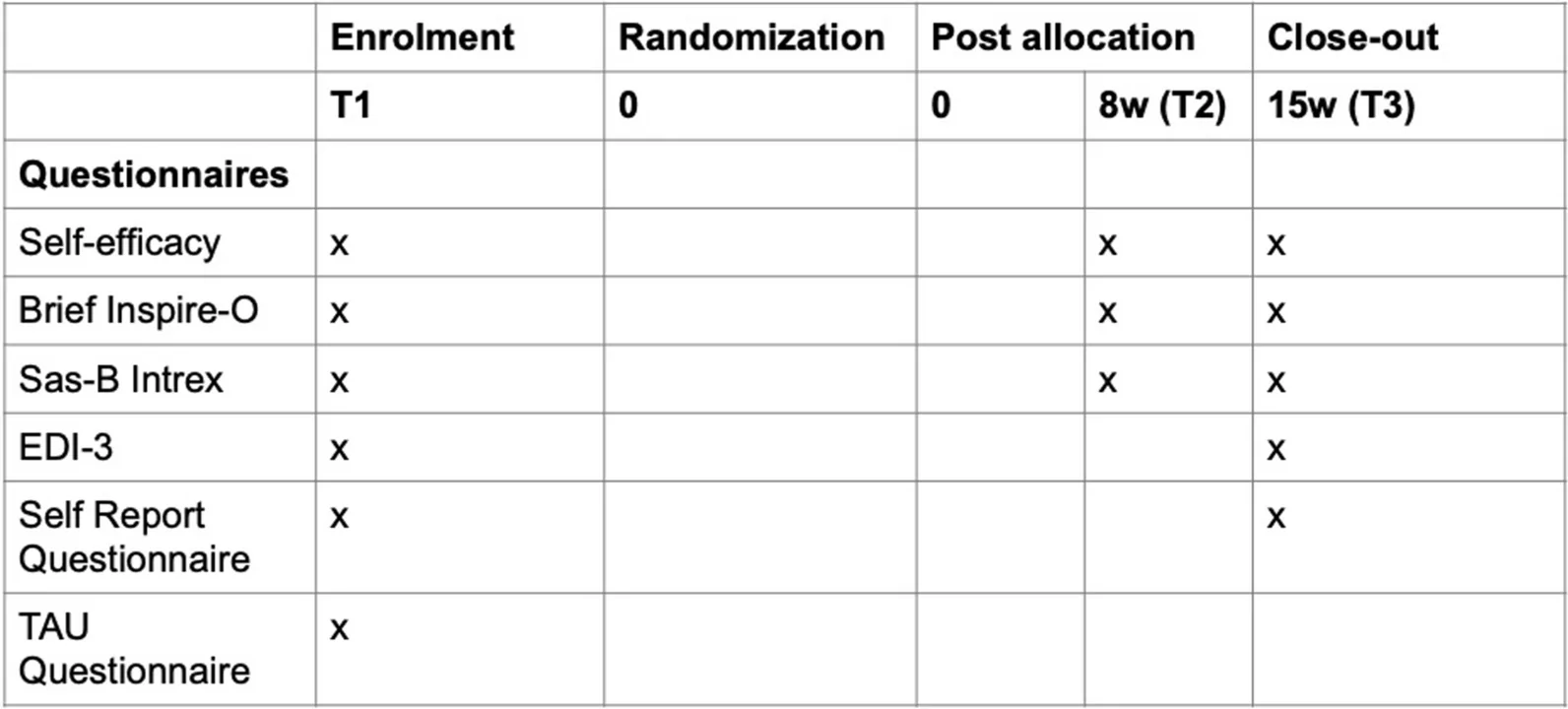
Overview of enrolment, interventions, and assessments. EDI-3, Eating Disorder Inventory, version 3; SASB, Structural Analysis of Social Behavior

Data on socio-demographic factors: age, gender, body mass index (BMI), ED diagnoses, prior ED treatment, duration and type of current ED treatment, psychiatric comorbidity, will be collected through patients’ medical records. In addition, data on occupation, children, social network, place and duration of treatment, ED symptoms and desired areas of personal development will be collected through a self-report questionnaire as stated below (T1), administered as an online questionnaire. Self-report measures will be collected at baseline (T1), and post-intervention 15 weeks after enrolment (T3). Patients will be asked to provide data during the intervention after 8 weeks (T2) and post intervention (T3) using questionnaires, distributed through their e-Boks, a secure digital mailbox for receiving and managing official documents and communications in Denmark [58]. Data are collected at following timepoints (Fig. 1):

At baseline (T1):

1. Self-report questionnaire created by the study authors and administered as an online questionnaire (Additional file 4: Appendix 4)
2. Socio-demographic data form patients’ case notes.
3. TAU questionnaire created to describe TAU across multiple sites (Additional file 3: Appendix 3)

At baseline (T1), 8 weeks (T2) and 15 weeks (T3):

4. Self-efficacy: The 6-item chronic disease self-efficacy scale is a 6-item self-report questionnaire, aimed to evaluate self-efficacy in terms of making decisions about one’s own life while suffering from chronic diseases [59]. It has been adapted by the authors to make it clinically appropriate to individuals suffering from ED (5-item SE_ED) (Additional file 5: Appendix 5).
5. Brief INSPIRE-O: is a 5-item questionnaire to measure recovery on five dimensions of personal recovery (i.e. connectedness with others, hope and optimism for the future, positive identity, meaning in existence, and empowerment) [60].
6. Structural Analysis of Social Behavior (SASB) Intrex Questionnaire is a 16-item questionnaire based on SASB model for self-image ratings where both positive and negative aspects of the self-image are assessed [61].

At baseline (T1) and 15 weeks (T3):

Eating Disorder Inventory, version 3 (EDI-3) [62] is a self-report questionnaire that consists of 91 items organized onto 12 primary scales, consisting of 3 eating-disorder-specific scales and 9 general psychological scales that are highly relevant to, but not specific to EDs. It also yields six composite scores, one that is eating disorder specific and five that are general integrative psychological constructs (i.e., Ineffectiveness, Interpersonal Problems, Affective Problems, Overcontrol, and General Psychological Maladjustment). We will focus on 2 of the composite scores: Ineffectiveness Composite (IC) and Interpersonal Problems Composite (IPC) [45].

### Statistical analysis

Socio-demographic data gathered at baseline will be summarized through descriptive statistics. Categorical variables will be expressed as frequencies and percentages, while continuous variables will be presented with mean, range, and standard deviation.

Changes in the primary outcome self-efficacy levels over time (baseline, after 8 and 15 weeks) will be modeled using linear mixed models. Participant-specific random intercepts will be included, as well as random slopes if these improve the model fit significantly. The fixed part of the model will consist of the intervention, time, and the treatment-time interaction. To assess the normality assumptions of residuals for both fixed and random effects, normal quantile–quantile plots will be employed. If deviations from normality are observed, sensitivity analyses will be conducted using non-parametric bootstrapping with 1,000 bootstrapping samples. A significance threshold of 0.05 will be applied. Assuming the dropout mechanism is missing at random (MAR), linear mixed models deal efficiently with missing values due to dropout using the maximum likelihood estimator. Therefore, all available data will used in an intention-to-treat approach (ITT).

The secondary outcomes INSPIRE-O and SAS-B will be analyzed in the same way as the primary outcome, while the model will be adjusted to include only the two measured time-points (baseline and 15 weeks) for the EDI-3, with focus on two composite scales: Ineffectiveness Composite (IC) and Interpersonal Problems Composite (IPC) from the EDI3.

To investigate missing data patterns and mechanisms, techniques such as descriptive statistics on missingness and analysis of missing data mechanisms (e.g., Little’s test) will be employed. Sensitivity analyses will be also conducted, investigating the complete case scenario and worst-case imputation to evaluate the robustness our imputed results.

A statistical analysis plan will be described before data analysis and posted on ClinicalTrials.gov before data analysis commences.

### Sample size calculation

Since Maze Out is a novel intervention, hardly any data are available that are useful for power-calculations. Our pilot study [29] showed a pre-post mean change score of 4 based on the same self-efficacy scale as used in the current study (SD = 1.5) at 8 weeks. We hypothesize that playing the game for 15 weeks will increase mean-score self-efficacy by 25%, compared to the TAU control group. Thus, with a power of 80% and alpha = 5%, and an assumed dropout rate of 20%, we need to include a total N = 94 (i.e., N = 47 per group) to be able to find medium effect size.

### Qualitative design

The qualitative exploration of Maze Out will involve individual and group interviews exploring the user experiences of using Maze Out and will seek insights into the detailed use of the SG and how it is experienced by patients with EDs as well as clinicians and significant others.

A subsample of patients from the RCT, in addition to a selection of clinicians and relatives, will be asked to participate in qualitative interviews, after playing Maze Out, with the purpose to investigate how Maze Out is experienced by its users. Purposive sampling will be used to select the participants. The purposeful sampling strategy aimed to identify participants who have played Maze Out a lot and those who played for a short time only or not at all. This strategy is chosen with the purpose to ensure that those who complete playing Maze Out and who do not complete it are both represented, and they will be included until data saturation.

Coding will be conducted using a concept map encompassing the topics of interview guide relating to the overall research questions. Codes will be grouped into semantic themes and organized according to the overall topics during the analysis process.

Nvivo 1.7.1 will be used to manage and organize qualitative data [63].

### Qualitative interviews

Three interview guides will be developed prior to the interview to explore users’ experiences of Maze Out, one for each group, with the purpose of allowing patients, clinicians, and family members to describe both the challenges and opportunities associated with the use of Maze Out. The interview guides will focus on overall experience of playing the SG, impact of the SG on relationships (e.g., health care staff and significant others), whether the SG gives insight into patients’ disorders, and if so, how. It will also capture participants’ reflections on whether the SG may have an impact on shame about ED symptoms and behaviours (Additional file 6: Appendix 6).

In addition to the qualitative data, quantitative data on the use of Maze Out will be included in the study. The quantitative data will be used to help the interpretation of the interviews in terms of a deeper understanding of the different perspectives depending on how the informant used the game.

### Qualitative data analysis

Thematic content analysis will be conducted using a realist methodological framework [64]. The analysis will be theoretical as it will be driven explicitly by the analysis from specific research questions, in contrast to questions evolving during the coding (i.e., an inductive approach). A theme in thematic content analysis represents both patterned responses and meanings that capture something important in relation to the research questions, but is not dependent on quantifiable measures [64]. In line with the theoretical approach, the identification of themes will be performed on a semantic level with grouping of explicit meanings and statements [65]. This implies that a unidirectional relationship is assumed between the statements of the participants and their meaning and/or motivations [66, 67]. The semantic patterns, or themes, will thus be summarized based on their surface meaning and the interpretation of these themes will be done focusing both on their explicit meaning but also on their implications and broader importance [68]. Example quotes will be provided across different themes.

Coding will be conducted using a concept map encompassing the topics of interview guide relating to the overall research questions. Codes will be grouped into semantic themes and organized according to the overall topics during the analysis process. Quantitative data on use of Maze Out from the back-end of Maze Out will be used to inform the qualitative data. For example, if there is a relation between the extend of playing and how it is experienced by the patient.

### Data triangulation

Qualitative and quantitative data will be triangulated to investigate the effect of confounders, and to examine the elements that may have an influence on the quantitative outcomes and exposure to the game. We expect that by doing so we will gain a deeper understanding of the mechanisms behind the use and effect of Maze Out and increase the validity and reliability of findings. A variety of sources (e.g., semi-structured interviews, self-report questionnaires and player statistics) will be used to extract data. Triangulating these data will aid interpretation of our results; for example, qualitative interviews will provide insight into the motivations or reasons on how many times a participant played the game.

## Discussion

The present study will investigate the effectiveness and experiences of an SG as a potential adjunct tool in the treatment of ED. Our hypothesis regarding the potential impact of Maze Out on patients with EDs is based on the results of a pilot experience and acceptance study [29], where we found that patients not only engaged in playing the SG, but that they also that experienced Maze Out as an opportunity to reflect on and gain insight into EDs and their associated behaviour patterns. The potential effects of the SG are, however, still unknown and may be more wide-ranging. To investigate this, we will explore the use of Maze Out using both qualitative and quantitative methodology. Triangulation of these methodological approaches will increase the validity and reliability of the findings and engender a deeper understanding of Maze Out as an adjunct tool.

Mobile phones are widely used in everyday life, and their use may predispose to a disconnection from the “real world”, on the other hand, people with eating disorders are usually afraid of being connected with others, their body sensations, and feelings. Taking this as a starting point Maze Out offers a known “safe place” where patients can practice those skills. Given the potential negative effects of excessive mobile phone use, Maze Out also offers an alternative to potentially destructive phone use (e.g. comparisons of body shape and exercise routines on Instagram) into considerably more positive use of mobile devices (e.g. working to resolve serious eating disorder symptoms).

### Innovative aspects

Previous findings suggest that SGs may be promising tools in the treatment of EDs. Maze Out is, not only the first SG for EDs to be played on a smartphone, but also the first SG coproduced by patients and clinicians. The high level of user input on its design and content can be argued to increase usability and potential impact as a therapeutic adjunct in ED treatment. Since effective treatment for ED is limited, tools that can function as helpful adjuncts in ED treatment are highly warranted.

### Challenges

In Denmark as in most societies, there are different treatment cultures and differences in how ED treatment is organized throughout the health care system. Since participants in the current study are recruited at sites with different treatment approaches, also in within the same treatment place, it will be impossible to capture the exact nature of TAU, or control for differences between treatment settings. The quantity of patients receiving same treatment in the same treatment place is so little that makes it impossible to stratify on TAU. We can’t therefore be sure that patients getting similar TAU are equally represented in the two arms. However, the broad recruitment of participants allows this study to examine whether adding Maze Out to TAU has real-world impact on patients’ self-efficacy, feelings of insecurity and their self-image with the variations that are seen across the health care system.

### Perspectives

Maze Out is a novel and theoretically grounded intervention that may have a significant effect on the healing processes of ED. Maze Out is expected to give patients with EDs the possibility to understand and manage everyday challenges when they need to, instead of waiting for the next appointment with the therapist. This will generate a sense of capacity about making important choices in life while living with this complex disorder. Furthermore, the use of the game by family members and therapists might help them to develop a common language about EDs and better understand the concrete challenges patients face at home and in social contexts.

We expect Maze Out to be experienced as fun to play, but at the same time to invite deep reflections on the personal challenges of an ED. This is done through an intervention that stimulates new forms or problem solving in a fresh, engaging, and relaxed manner. Moreover, we hope that the results of this study will enable the opportunity to create a handbook on the specific use of Maze Out, so that Maze Out can be implemented effectively across different healthcare systems.

### Current status of the project

We are currently recruiting patients, clinicians, and family members. Recruitment is expected to be concluded on December the 1st 2023.

## Supplementary Information

**Additional file 1.** Patients participants in the co-production of Maze Out.

**Additional file 2.** Mission "Say what you want".

**Additional file 3.** Treatment as usual questionnaire (for patients).

**Additional file 4.** Self-report questionnaire.

**Additional file 5.** Self-efficacy scale: 5-item SE_ED.

**Additional file 6.** Interview guide for patients.

## Data Availability

The original contributions presented in the study are included in the article/Supplementary Material, further inquiries can be directed to the corresponding author.
