# Correction: “Maze Out”: a study protocol for a randomised controlled trial using a mix methods approach exploring the potential and examining the effectiveness of a serious game in the treatment of eating disorders

**DOI:** 10.1186/s40337-026-01760-1

**Published:** 2026-09-03

**Authors:** Maria Mercedes Guala, Aida Bikic, Kim Bul, David Clinton, Anna Mejldal, Helene Nygaard Nielsen

**Affiliations:** 1 Psychiatric Research Unit, Institute of Clinical Research, University of Southern Denmark, J.B. Winsløws Vej 18, Odense, 5000 Denmark; 2 Department of Regional Health Research, Faculty of Health, University of Southern Denmark, Odense, Denmark; 3 Centre for Intelligent Healthcare, Coventry University, Coventry, UK; 4 Department of Medical Epidemiology and Biostatistics (MEB), Centre for Eating Disorders Innovation (CEDI), Karolinska Institute, Stockholm, Sweden; 5 Open Patient Data Explorative Network, Odense University Hospital, Odense, Denmark; 6 Child and Adolescent Psychiatric Services Southern Jutland, Region of Southern Denmark, Aabenraa, Denmark

**Correction: Journal of Eating Disorders (2024) 12:35**

**https://doi.org/10.1186/s40337-024-00985-2**

Following the publication of the original article, the author(s) reported typo in the name of the fifth author.

- Incorrect: Anna Mejdal.
- Correct: Anna Mejldal.

The original article has been corrected.

