# Supplementary material for: “Maze Out”: a study protocol for a randomised controlled trial using a mix methods approach exploring the potential and examining the effectiveness of a serious game in the treatment of eating disorders
Source: J Eat Disord. 2024 Mar 1;12:35. doi: 10.1186/s40337-024-00985-2 (PMC10908122; doi:10.1186/s40337-024-00985-2)
Supplement: Supplementary file 1 — Additional file 1. Patients participants in the co-production of Maze Out. [file 40337_2024_985_MOESM1_ESM.docx]

Appendix 1

| Participants |  |
| --- | --- |
| Patients | 4 |
| Age | 19, 25, 28, 35 |
| *ED diagnosis:*  Anorexia nervosa  Bulimia nervosa  Eating disorder not otherwise specified | 2  1  1 |
| *Length of ED treatment:*  In the middle of treatment  At the end of the treatment’  Over 5 years  Finished treatment | 1  1  1  1 |
| *Academic background:*  High school  Shorter education  Medium-length higher education | 2  1  1 |
