# Supplementary material for: “Maze Out”: a study protocol for a randomised controlled trial using a mix methods approach exploring the potential and examining the effectiveness of a serious game in the treatment of eating disorders
Source: J Eat Disord. 2024 Mar 1;12:35. doi: 10.1186/s40337-024-00985-2 (PMC10908122; doi:10.1186/s40337-024-00985-2)
Supplement: Supplementary file 2 — Additional file 2. Mission "Say what you want". [file 40337_2024_985_MOESM2_ESM.pdf]

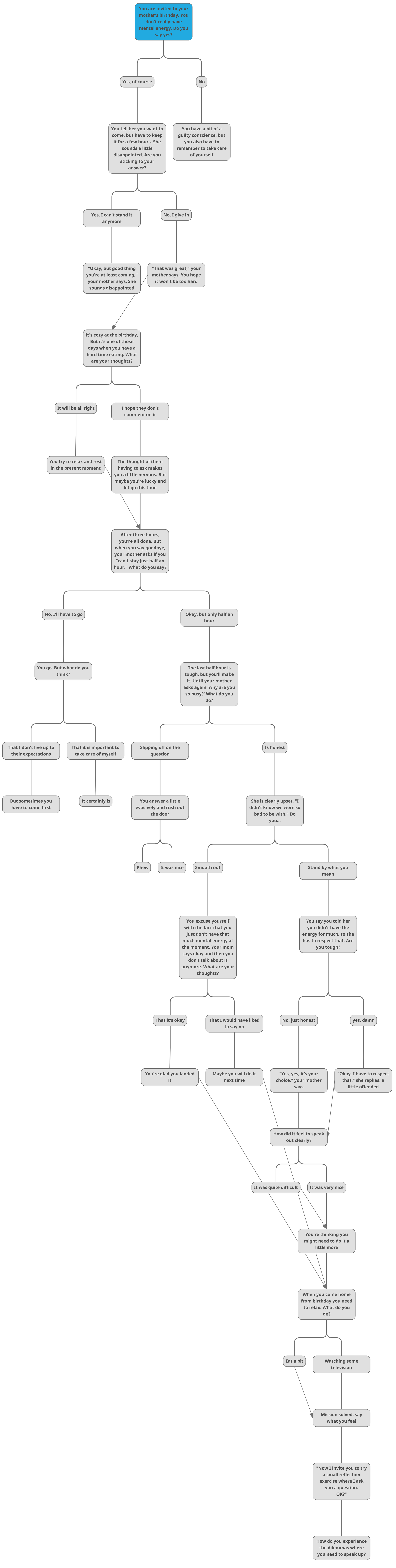

You are invited to your mother's birthday. You don't really have mental energy. Do you say yes?
Yes, of course
No
You tell her you want to come, but have to keep it for a few hours. She sounds a little disappointed. Are you sticking to your answer?
You have a bit of a guilty conscience, but you also have to remember to take care of yourself
Yes, I can't stand it anymore
No, I give in
"Okay, but good thing you're at least coming," your mother says. She sounds disappointed
"That was great," your mother says. You hope it won't be too hard
It's cozy at the birthday. But it's one of those days when you have a hard time eating. What are your thoughts?
It will be all right
I hope they don't comment on it
You try to relax and rest in the present moment
The thought of them having to ask makes you a little nervous. But maybe you're lucky and let go this time
After three hours, you're all done. But when you say goodbye, your mother asks if you "can't stay just half an hour." What do you say?
No, I'll have to go
Okay, but only half an hour
You go. But what do you think?
The last half hour is tough, but you'll make it. Until your mother asks again 'why are you so busy?' What do you do?
That I don't live up to their expectations
That it is important to take care of myself
Slipping off on the question
Is honest
But sometimes you have to come first
It certainly is
You answer a little evasively and rush out the door
She is clearly upset. "I didn't know we were so bad to be with." Do you...
Phew
It was nice
Smooth out
Stand by what you mean
You excuse yourself with the fact that you just don't have that much mental energy at the moment. Your mom says okay and then you don't talk about it anymore. What are your thoughts?
You say you told her you didn't have the energy for much, so she has to respect that. Are you tough?
That it's okay
That I would have liked to say no
No, just honest
yes, damn
You're glad you landed it
Maybe you will do it next time
"Yes, yes, it's your choice," your mother says
"Okay, I have to respect that," she replies, a little offended
How did it feel to speak out clearly?
It was quite difficult
It was very nice
You're thinking you might need to do it a little more
When you come home from birthday you need to relax. What do you do?
Eat a bit
Watching some television
Mission solved: say what you feel
"Now I invite you to try a small reflection exercise where I ask you a question. OK?"
How do you experience the dilemmas where you need to speak up?
