# Supplementary material for: “Maze Out”: a study protocol for a randomised controlled trial using a mix methods approach exploring the potential and examining the effectiveness of a serious game in the treatment of eating disorders
Source: J Eat Disord. 2024 Mar 1;12:35. doi: 10.1186/s40337-024-00985-2 (PMC10908122; doi:10.1186/s40337-024-00985-2)
Supplement: Supplementary file 3 — Additional file 3. Treatment as usual questionnaire (for patients). [file 40337_2024_985_MOESM3_ESM.docx]

# Appendix 3

## Treatment as usual questionnaire (for patients)

1. How long have you been in treatment for an eating disorder? (current treatment episode)

- 0-2 months
- 3-5 months
- 6-11 months
- 1-2 years
- More than 2 years

1. Where do you go for treatment?

- Free text

1. What kind of treatment? (it is possible to select several answers)

- Outpatient Group therapy
- Outpatient Individual therapy
- Inpatient
- Other

The questionnaire is supplemented with information from/about the treatment center, such as type of offered treatment ( MBT, CBT, type of physiotherapy approach).
