# Supplementary material for: “Maze Out”: a study protocol for a randomised controlled trial using a mix methods approach exploring the potential and examining the effectiveness of a serious game in the treatment of eating disorders
Source: J Eat Disord. 2024 Mar 1;12:35. doi: 10.1186/s40337-024-00985-2 (PMC10908122; doi:10.1186/s40337-024-00985-2)
Supplement: Supplementary file 4 — Additional file 4. Self-report questionnaire. [file 40337_2024_985_MOESM4_ESM.docx]

# Appendix 4:

All questions are asked at baseline and only from 3 to 6 are asked again after 15 weeks.

## Self-report questionnaire

1. What is your current occupation?

- Full or part-time study
- full-time work
- part-time work
- jobless
- on sick leave
- stay-at-home
- unemployment benefit
- pensioner
- other

1. Are you the parent of one or more children?

- yes
- no

1. Who are you in regular contact with? (multiple answers are possible)

- parents
- partner/spouse
- children
- friends
- contact persons
- others

1. How often you have contact with your network?

- daily
- weekly
- 1-2 times per month
- less often

1. Do you have the following symptoms?

- vomiting
- overexercise
- slimming pills or laxatives
- overeating

1. How important is it for you to develop in the following areas?

- have a healthier relationship with food
- get a healthier relationship with exercise
- stop vomiting
- deal with binge eating
- improve at expressing my feelings
- be better at setting boundaries
- improve respect for my own needs and learn to stop controlling.
- improve their participation in social contexts
- improve their participation in social contexts
