# Supplementary material for: “Maze Out”: a study protocol for a randomised controlled trial using a mix methods approach exploring the potential and examining the effectiveness of a serious game in the treatment of eating disorders
Source: J Eat Disord. 2024 Mar 1;12:35. doi: 10.1186/s40337-024-00985-2 (PMC10908122; doi:10.1186/s40337-024-00985-2)
Supplement: Supplementary file 5 — Additional file 5. Self-efficacy scale: 5-item SE_ED. [file 40337_2024_985_MOESM5_ESM.docx]

# Appendix 5

Self-Efficacy for Managing Chronic Disease, a 6-item Scale adapted and slightly modified by the author to evaluate self-efficacy in terms of making decisions about one’s own life when having an ED.

Maze Out focuses on helping patients in their path to be more aware of inner experiences, to rely on feelings, thoughts, and bodily sensations to guide behaviour and the forms of progress defined by the patients that participate in the co-production of the game. In the co-production process patients focused on their experiences or/ and hopes of living a satisfying life even with the limitations caused by ED.

No existing scale covered these aspects of self-efficacy in an ED population. The scale that was closest to what we aim to look for is the Self-Efficacy Scale for Managing Chronic Disease (SECD) [1]. The SECD scale was adapted in two domains: the number of questions and the response options. The 6^th^ question in SECD is about medication and was omitted. The reason of taking out question 6 was that medication is only used to treat comorbidity in ED. The SECD uses a Likert scale from 1 to 10 as response options. In our study we have used verbal labels instead of numbers and reduced the options from 10 to 7. Since the population of this study is characterised by difficulties making decisions, we have attempted to make response options clear and the number of alternatives manageable.

## Self-efficacy scale

- How confident do you feel that you can…

1. Manage the fatigue caused by your disease from interfering with the things you want to do?

- completely uncertain, very uncertain, uncertain, a bit uncertain, somewhat certain, certain, very certain, definitely

1. Keep the physical discomfort or pain of your disorder from interfering with the things you want to do?

- completely uncertain, very uncertain, uncertain, a bit uncertain, somewhat certain, certain, very certain, definitely

1. Keep the emotional distress caused by your disease from interfering with the things you want to do?

- completely uncertain, very uncertain, uncertain, a bit uncertain, somewhat certain, certain, very certain, definitely

1. Keep any other symptoms or health problems you have from interfering with the things you want to do?

- completely uncertain, very uncertain, uncertain, a bit uncertain, somewhat certain, certain, very certain, definitely

1. The different tasks and activities needed to manage your health condition so as to reduce your need to seek help?

- completely uncertain, very uncertain, uncertain, a bit uncertain, somewhat safe, safe, very safe, definitely

1. Lorig KR, Sobel DS, Ritter PL, Laurent D, Hobbs M. Effect of a self-management program on patients with chronic disease. Effective clinical practice: ECP. 2001;4(6):256-62.
