# Supplementary material for: “Maze Out”: a study protocol for a randomised controlled trial using a mix methods approach exploring the potential and examining the effectiveness of a serious game in the treatment of eating disorders
Source: J Eat Disord. 2024 Mar 1;12:35. doi: 10.1186/s40337-024-00985-2 (PMC10908122; doi:10.1186/s40337-024-00985-2)
Supplement: Supplementary file 6 — Additional file 6. Interview guide for patients. [file 40337_2024_985_MOESM6_ESM.docx]

# Appendix 6

# Interview guide: patients

This interview guide is only a guide and will be used as a check list for the interviewer and not intended to be followed word by word. The interview will be conducted as an open dialog where the patient will have the possibility to explain in depth the aspects of the questions he or she wants to.

## Register the patient's:

• Diagnosis

• Age

• Treatment form

• Duration of illness since debut

1. Can you tell me what got you interested in Maze Out? (focus not only on why she agreed to participate but also what made her play to such an extent)

2. Can you describe your experience of the game? Or: Can you tell me how you experienced the game? (focus on giving space and spotting what the patient is preoccupied with in relation to the game, so that the questions can be adjusted accordingly)

3. From your perspective, what made you play the game? (focus on game function)

4. What do you think you got out of playing? Immediately after and now after a while? (focus on motivation and what function the game could have in everyday life)

5. Do you think the game has influenced the way you understand yourself/that of suffering from a eating disorder? How? (focus on self-insight)

6. Do you think that Maze Out can affect the feelings one can have when suffering from eating disorders? (if so, encourage to talk more specifically about her own experience).

7. From your perspective, what do you think the game has brought you that you don't experience in the treatment you receive? (focus on: accessibility, that it's a game...)

8. Has the game influenced how you have spoken to your relatives or treated the fact that you suffer from an eating disorder? (If yes, in what way)

9. Is there anything else you think is important for us to know regarding further use and development of the game?
